# P-1797. Implementation of a Handshake Stewardship Pilot for Inpatient General Medicine Teams at an Academic Medical Center

**DOI:** 10.1093/ofid/ofae631.1960

**Published:** 2025-01-29

**Authors:** Joseph Stromberg, Ashley H Marx, Nikolaos Mavrogiorgos

**Affiliations:** UNC Medical Center, Durham, North Carolina; University of North Carolina Medical Center, Chapel Hill, North Carolina; University of North Carolina Medical Center, Chapel Hill, North Carolina

## Abstract

**Background:**

Handshake stewardship is an approach to antimicrobial stewardship that relies on in-person discussions between ASP teams and primary inpatient teams. This model has been implemented in pediatric and intensive care settings, and outcomes have been described in observational studies. In November 2023, UNCMC implemented a handshake stewardship pilot with crossover design for select inpatient general medicine teams.

**Figure:**
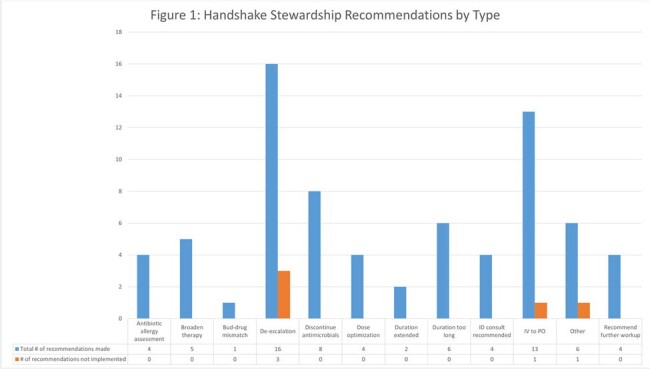

**Methods:**

Three pairs of matched inpatient general medicine teams at UNCMC were selected, with one team from each pair included in a three month intervention period. After three months, the teams were switched. The intervention consisted of twice weekly meetings of ASP physician and/or pharmacist and primary teams, during which antimicrobial regimens of all patients were reviewed except those with an ID consult. The intervention was delivered via in-person meetings for the two pairs of teams located at UNCMC, and via teleconference for a pair of teams located on a separate campus. The remote teams withdrew from the pilot during the intervention period. Recommendations made were catalogued as iVents in the Epic EMR and electronic surveys were circulated to primary team providers.

**Figure:**
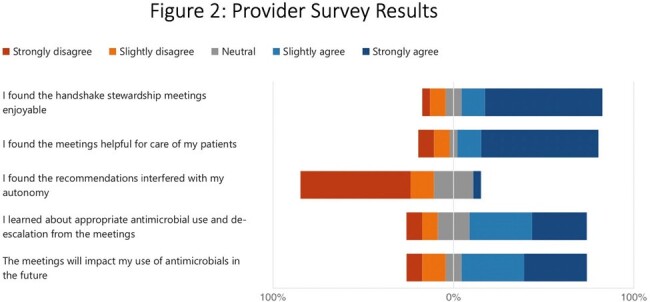

**Results:**

287 patients were reviewed across 95 meetings to date, yielding 73 recommendations. Common themes included narrowing coverage and transitioning to oral agents, although in some cases ASP teams recommended broadening therapy or a formal ID consult (Figure 1). 93% of recommendations were implemented. Survey data from 23 participants showed positive attitudes (Figure 2), with many valuing the opportunity to discuss patients that didn’t require a formal ID consult. Survey feedback from the virtual team included frustration with cessation of in-person ID consults at the site (unrelated to this pilot).

**Conclusion:**

Handshake stewardship with in-person meetings has been well-received at UNCMC based on logged interventions and provider surveys, although the virtual component was unsuccessful. A planned crossover comparison between teams and paired controls will be helpful in assessing whether this has had an impact on antimicrobial use. Facilitating successful virtual handshake ASP will require improved coordination of meeting times and ensuring adequate access to in-person ID consults.

**Disclosures:**

**All Authors**: No reported disclosures

